# Serum TERT C228T is an important predictor of non-viral liver cancer with fatty liver disease

**DOI:** 10.1007/s12072-022-10313-y

**Published:** 2022-03-20

**Authors:** Norio Akuta, Yusuke Kawamura, Fumitaka Suzuki, Mariko Kobayashi, Yasuji Arase, Satoshi Saitoh, Nozomu Muraishi, Shunichiro Fujiyama, Hitomi Sezaki, Tetsuya Hosaka, Masahiro Kobayashi, Yoshiyuki Suzuki, Kenji Ikeda, Hiromitsu Kumada

**Affiliations:** 1 Department of Hepatology, Toranomon Hospital and Okinaka Memorial Institute for Medical Research, 2-2-2 Toranomon, Minato-ku, Tokyo, 105-8470 Japan; 2 Liver Research Laboratory, Toranomon Hospital, Tokyo, Japan

**Keywords:** *TERT* promoter, cfDNA, AFP, PIVKAII, Etiology, HBV, HCV, FLD, NAFLD, AFLD

## Abstract

**Background:**

Molecular therapies and precision medicine are expected to be developed for liver cancer based on the diagnosis of DNA somatic alterations. However, it remains unclear whether *TERT* promoter mutation (TERT C228T) in serum cfDNA is useful for the diagnosis of liver cancer with non-viral fatty liver disease (FLD).

**Methods:**

This retrospective cohort study examined 258 Japanese patients who had a confirmed diagnosis of primary liver cancer. We investigated the factors associated with TERT C228T and abnormal levels of liver cancer-specific tumor markers (AFP and PIVKAII) in serum samples.

**Results:**

Multivariate analysis identified the etiology of FLD, vascular invasion, and non-cirrhosis as determinants of TERT C228T-positive liver cancer. Rates of positive TERT C228T in FLD were significantly higher than those of HBV and HCV. Conversely, rates of abnormal AFP in FLD were significantly lower than those of HBV and HCV. Viral suppression of HBV/HCV and alcohol intake did not affect TERT C228T, but AFP was significantly reduced by viral suppression. The rates of positive TERT C228T were significantly lower in HCV patients with viral clearance than those of FLD patients.

**Conclusion:**

Our results highlight the importance of serum TERT C228T for the detection of non-viral FLD-related liver cancer. TERT C228T is a tumor marker that might not be influenced by inflammation.

**Supplementary Information:**

The online version contains supplementary material available at 10.1007/s12072-022-10313-y.

## Introduction

The development of molecular therapies and precision medicine is expected for liver cancer based on the diagnosis of DNA somatic alterations. Genomic studies have identified telomerase reverse transcriptase (*TERT*), tumor protein p53, and catenin beta 1 as the most frequently mutated genes in liver cancer [1–3]. *TERT* promoter mutation is the most frequent genetic alteration in liver cancer [3, 4]. Furthermore, two hotspots of *TERT* promoter mutations, C228T and C250T, have been detected in 94.7% and 5.3% of patients with identified mutations, respectively. Thus, a stronger impact of C228T than C250T is presumed in liver cancer [5]. Recent reports show that NAFLD-related liver cancer might be less responsive to immune checkpoint inhibitors [6]. Furthermore, *Wnt/CTNNB1* mutation (one type of DNA somatic alteration) might be a biomarker that could predict resistance to such therapies [7].

Our recent report highlighted the better performance of TERT C228T in serum cfDNA than AFP and PIVKAII in the early diagnosis of primary liver cancer in patients with non-alcoholic fatty live disease (NAFLD) [8]. AUROC, sensitivity, specificity, PPV, and NPV of TERT C228T were 0.812, 63.9%, 95.2%, 95.8%, and 60.6% in predicting NAFLD-related liver cancer, respectively. Those of PIVKAII positivity were 0.735, 36.1%, 66.7%, 65.0%, and 37.8%, respectively. Those of AFP positivity were 0.507, 36.1%, 66.7%, 65.0%, and 37.8%, respectively. Namely, in predicting NAFLD-related liver cancer, kappa coefficients were 0.528, 0.389, and 0.024 in TERT C228T, PIVKAII positivity, and AFP positivity, respectively [8]. However, it remains unclear whether serum TERT C228T is useful for the diagnosis of non-viral fatty liver disease (FLD)-related liver cancer, which has had an increasing trend recently [9]. Thus, the purpose of the present retrospective study was to determine the clinical and histopathological factors associated with TERT C228T in serum samples, as well as to investigate the useful marker for the diagnosis of FLD-related liver cancer.

## Materials and methods

### Patients

This retrospective cohort study examined 258 Japanese patients. The patients were confirmed to have a diagnosis of primary liver cancer for the first time through imaging studies between 1984 and 2020 at Toranomon Hospital. There were 117 patients who also underwent surgical resection and had a confirmed diagnosis of liver cancer with histopathological examination. Table 1 summarizes the characteristics of the 258 patients. We investigated the clinical and histopathological factors associated with TERT C228T in serum samples obtained at the first diagnosis of primary liver cancer.

**Table 1:** Characteristics of 258 patients with liver cancer

Overall subjects (*n* = 258)	
Demographic data	
Gender, males/females, *n*	171/87
Age, years	67 (34–88)
Body mass index, kg/m^2^	24.3 (13.6–41.6)
Type 2 diabetes mellitus, absence/presence, *n*	179/79
Etiology	
HBV/HCV/FLD (NAFLD/AFLD)	90/96/72 (52/20)
Antiviral therapy for HBV or HCV	
NUCs for HBV, absence/presence, *n*	57/33
DAAs for HCV, absence/presence (non-SVR/SVR), *n*	30/66 (10/56)
Laboratory data	
Serum aspartate aminotransferase, U/L	36 (10–207)
Serum alanine aminotransferase, U/L	32 (7–204)
Platelet count, × 10^3^/mm^3^	132 (17–457)
Albumin, g/dL	3.8 (2.1–4.9)
Total bilirubin, mg/L	0.9 (0.2–12.1)
Prothrombin activity, %	86.0 (33.1–113.3)
Fasting plasma glucose, mg/dL	101 (69–392)
Fib-4 index	3.34 (0.55–28.7)
Child–Pugh classification, A/B/C/unknown, *n*	213/36/8/1
AFP, μg/L	10 (1–16,659)
PIVKAII, AU/L	26 (1–157,050)
TERT C228T, negative/positive	166/92
Tumor characteristics, based on the image findings	
Maximum tumor diameter, mm	20 (6–150)
Number of tumors, 1/2/3/4 or more/unknown, *n*	199/41/12/5/1
Macrovascular invasion, absence/presence, *n*	246/12
Extrahepatic metastasis, absence/presence, *n*	258/0
BCLC stage, 0/A/B/C/D/unknown, *n*	60/167/14/7/8/2
Patients, who were evaluated histopathological findings (*n* = 117)	
Tumor tissue	
Number of tumors, 1/2/3, n	106/10/1
Maximum tumor diameter, mm	23 (4–140)
Organization type	
Well-differentiated HCC, not contain/contain/unknown, *n*	72/43/2
Moderately-differentiated HCC, not contain/contain/unknown, *n*	12/103/2
Poorly-differentiated HCC, not contain/contain/unknown, *n*	95/20/2
Cholangiocellular carcinoma, not contain/contain/unknown, *n*	110/5/2
Formation of capsule, absence/presence/unknown, *n*	27/83/7
Infiltration to capsule, absence/presence/unknown, *n*	33/63/21
Septal formation, absence/presence/unknown, *n*	32/73/12
Serosal infiltration, absence/presence/unknown, *n*	103/2/12
Vascular invasion	
vp, absence/presence/unknown, *n*	77/38/2
vv, absence/presence/unknown, *n*	102/7/8
va, absence/presence/unknown, *n*	104/0/13
Bile duct invasion, absence/presence/unknown, *n*	98/2/17
Intrahepatic metastasis, absence/presence/unknown, *n*	91/3/23
Peritoneal dissemination, absence/presence/unknown, *n*	81/0/36
Non tumor tissue	
Fibrosis stage, 0/1/2/3/4/unknown, *n*	1/17/14/31/53/1

Data are number of patients or median (range) values

*AFP* alpha-fetoprotein, *AFLD* alcoholic fatty liver disease, *BCLC* Barcelona Clinic Liver Cancer, *DAAs* direct-acting antivirals, *FLD* fatty liver disease, *HBV* hepatitis B virus, *HCV* hepatitis C virus, *NAFLD* non-alcoholic fatty liver disease, *NUCs* nucleos(t)ide analogues, *PIVKAII* des-γ-carboxyprothrombin, *SVR* sustained virological response

The following criteria were used to select 90 patients with HBV-related liver cancer: (1) a positive test for HBV surface antigen (Chemiluminescent Enzyme Immunoassay, Abbott Laboratories, Tokyo, Japan), (2) a negative test for HCV antibody by third-generation enzyme immunoassay (Chiron Corp, CA, USA), (3) history of mild to moderate alcohol intake (estimated lifetime cumulative alcohol intake of < 500 kg), and (4) confirmed lack of hemochromatosis, Wilson disease, primary biliary cholangitis, and autoimmune liver disease. There were 57 patients who did not receive antiviral therapy (nucleos(t)ide analogues [NUCs]) and were diagnosed with liver cancer. Liver cancer was detected in the other 33 patients, regardless of the achievement of viral suppression under NUCs.

The following criteria were used to select 96 patients with HCV-related liver cancer: (1) a positive test for HCV antibody and HCV RNA by quantitative analysis before antiviral therapy (direct-acting antivirals [DAAs]), (2) negative test for HBV surface antigen, (3) history of mild to moderate alcohol intake, and (4) confirmed lack of hemochromatosis, Wilson disease, primary biliary cholangitis, and autoimmune liver disease. Sustained virological response (SVR) regarded as HCV clearance was defined as a negative HCV RNA result at 12 weeks after the cessation of DAAs according to the COBAS TaqMan HCV test (Roche Diagnostics, Tokyo, Japan). There were 30 patients who did not receive DAAs and were diagnosed with liver cancer. Liver cancer was detected in 10 patients after the diagnosis of non-SVR by DAAs, and in the other 56 patients, it was detected after the diagnosis of SVR by DAAs.

The following criteria were used to select 72 patients with FLD-related liver cancer: (1) histopathological changes of steatosis in at least 5% of hepatocytes, (2) negative test for HBV surface antigen and HCV antibody, and (3) a confirmed lack of viral hepatitis, drug-induced liver disease, hemochromatosis, α-1-antitrypsin deficiency, Wilson disease, primary biliary cholangitis, autoimmune liver disease, and systemic autoimmune diseases (e.g., systemic lupus erythematosus or rheumatoid arthritis). There were 52 patients with NAFLD, which was defined by an upper limit of alcohol intake of 30 g/day in males and 20 g/day in females [9]. There were 20 patients with alcoholic fatty liver disease (AFLD), which was defined as alcohol intake in excess of the upper limit.

The Human Ethics Review Committee at Toranomon Hospital approved the protocol of the study. Signed informed consent forms were obtained from each of the patients at the time of liver histological diagnosis. The study complied with the International Conference on Harmonization Guidelines for Good Clinical Practice (E6) and the 2013 Declaration of Helsinki.

### Diagnosis of liver cancer

The diagnosis of liver cancer in all 258 patients was confirmed by imaging studies, including abdominal ultrasound (US), dynamic computed tomography (CT), and magnetic resonance imaging (MRI). For the 117 patients (45.3%) who underwent surgical resection, the diagnosis of liver cancer was confirmed with histopathological examination. The tumor characteristics were evaluated according to the Barcelona Clinic Liver Cancer (BCLC) staging [10].

### Clinical parameters

A normal level of AFP was defined as 10 μg/L or less, and that of PIVKAII was 40 AU/L or less. The Fib-4 index was used as a parameter for the progression of fibrosis and was calculated as follows: [age (year) × AST (IU/L)]/[platelet count (10^9^/L) × √ALT (IU/L)] [11].

### Assessment of *TERT* promoter mutation by wild-type blocking PCR

Our group recently developed a highly sensitive method for the detection of *TERT* promotor mutation using wild-type blocking PCR (WTB-PCR), combined with Sanger sequencing, and demonstrated its clinical usefulness for early prediction of liver cancer, by measuring TERT C228T in serum cfDNA [12]. The sequencing analysis of WTB-PCR product demonstrated a detection limit in excess of 0.7% Mutant-type DNA in the background of Wild-type DNA [12]. Thus, in the present study we serially examined the relationship between liver cancer and TERT C228T in serum cfDNA by WTB-PCR.

After withdrawal of blood samples, serum was frozen at – 80 °C within 4 h of collection then thawed just before analysis. The genome DNA was extracted from 1,000 μL of serum with QIAamp® Circulating Nucleic Acid Kit (Qiagen, Tokyo), and the nucleotide sequences were determined by direct sequencing. The primers used were TERT promoter F (5´-CAGCGCTGCCTGAAACTC-3´; nucleotides 1,295,151–1,295,168 on chromosome 5) and TERT promoter R2 (5´-GGCCGATTCGACCTCTCT-3´; nucleotides 1,295,528–1,295,511 on chromosome 5). The genome sequence of 378 nucleotides was determined. The 228-LNA (5´-gcccagcccCCTccgggccct-3´; capital letters indicate LNA) was used as the blocking oligonucleotide for *TERT* promoter at position 228 (TERT228). WTB-PCR master mix was prepared using 12.5 µL 2 × buffer, 5 µL dNTPs, 1 µL forward primer, 1 µL reverse primer, 1 µL blocking oligonucleotide for TERT228, 0.5 µL KOD SYBR^®^ qPCR Mix (Toyobo Co., Osaka, Japan), and 3 µL double-distilled H_2_O to create a final solution volume of 24 µL per reaction. Of this, 1 µL was used for genomic DNA. First denaturation was performed at 94 ºC for 2 min, and 40 cycles of amplifications were performed as follows: denaturation for 10 s at 98 ºC, annealing of primers for 30 s at 62 ºC followed by 5 s at 72 ºC, extension for 30 s at 68 ºC, and final extension was performed at 68 ºC for 7 min. The PCR-amplified DNA was purified after agarose gel electrophoresis and then used for direct sequencing. The latter was conducted using the dye terminator method. Dideoxynucleotide termination sequencing was performed using the Big Dye^®^ Terminator Cycle Sequencing kit (Life Technologies, Tokyo). We defined TERT C228T “positive” samples as those with mutant peak detected at position 228 (228 T), based on the electropherograms in sequencing [8, 12].

### Statistical analysis

Non-parametric tests were used to compare variables between groups, including the chi-squared test, Fisher’s exact probability test, and Mann–Whitney *U* test. Univariate and multivariate logistic regression analyses were used to determine the independent predictive factors associated with TERT C228T-positive liver cancer. The parameters in Table 1 that indicated strong correlations with other parameters were considered confounding factors and excluded from the univariate and multivariate analyses. Thus, the parameters shown in Tables 2, 3 were used for the analysis of the predictive factors.

**Table 2:** Clinical factors associated with TERT C228T-positive liver cancer

Factors	Category	Univariate analysis	Multivariate analysis		
		p*	Odds ratios	(95% confidence interval)	p*
Demographic data					
Gender	Male				
	Female	0.414			
Body mass index, kg/m^2^	< 25.0				
	≥ 25.0	0.047			
Type 2 diabetes mellitus	Absence				
	Presence	0.121			
Etiology	HBV		1		
	HCV	0.751**	1.066	(0.568–2.002)	0.841
	FLD	0.009***	2.346	(1.223–4.500)	0.010
Laboratory data					
Fib-4 index	< 3.25				
	≥ 3.25	0.195			
AFP, μg/L	< 11				
	≥ 11	0.897			
PIVKAII, AU/L	< 41				
	≥ 41	0.577			
Tumor characteristics					
Maximum tumor diameter, mm	< 20				
	≥ 20	0.359			
Number of tumors	1				
	≥ 2	0.351			
Macrovascular invasion	Absence				
	Presence	0.760			
Extrahepatic metastasis	Absence				
	Presence	1.000			
BCLC stage	0, A				
	B, C, D	0.154			

Normal level of AFP was defined as 10 μg/L or less, and that of PIVKAII was 40 AU/L or less

*AFP* alpha-fetoprotein, *BCLC* Barcelona Clinic Liver Cancer, *FLD* fatty liver disease, *HBV* hepatitis B virus, *HCV* hepatitis C virus, *PIVKAII* des-γ-carboxyprothrombin

^*^Uni- and multivariate logistic regression analyses were applied to identify clinical factors associated with TERT C228T positive. Variables that achieved statistical significance (*p* < 0.05) and marginal significance (*p* < 0.1) on univariate analysis were entered into multiple logistic regression analysis to identify significant independent factors. **HBV vs. HCV, ***HBV vs. FLD

**Table 3:** Histopathological factors associated with TERT C228T-positive liver cancer

Factors	Category	Univariate analysis	Multivariate analysis		
		p*	Odds ratios	(95% confidence interval)	p*
Tumor tissue					
Number of tumors	1	0.190			
	≥ 2				
Maximum tumor diameter, mm	< 20	0.693			
	≥ 20				
Organization type					
Well-differentiated HCC	Not contain	0.549			
	Contain				
Moderately-differentiated HCC	Not contain	1.000			
	Contain				
Poorly-differentiated HCC	Not contain	0.442			
	Contain				
Cholangiocellular carcinoma	Not contain	0.346			
	Contain				
Formation of capsule	Absence	0.258			
	Presence				
Infiltration to capsule	Absence	0.506			
	Presence				
Septal formation	Absence	0.824			
	Presence				
Serosal infiltration	Absence	1.000			
	Presence				
Vascular invasion					
vp	Absence		1		
	Presence	0.097	2.472	(1.057–5.784)	0.037
vv	Absence				
	Presence	1.000			
va	Absence				
	Presence	1.000			
Bile duct invasion	Absence				
	Presence	0.127			
Intrahepatic metastasis	absence				
	presence	0.296			
Peritoneal dissemination	Absence				
	Presence	1.000			
Non tumor tissue					
F ibrosis stage	3, 4		1		
	0, 1, 2	0.047	3.774	(1.565–9.091)	0.003

*AFP* alpha-fetoprotein, *PIVKAII* des-γ-carboxyprothrombin, *HCC* hepatocellular carcinoma

^a^Uni- and multivariate logistic regression analyses were applied to identify histopathological factors associated with TERT C228T positive. Variables that achieved statistical significance (*p* < 0.05) and marginal significance (*p* < 0.1) on univariate analysis were entered into multiple logistic regression analysis to identify significant independent factors

Each variable was transformed into categorical data consisting of two simple ordinal numbers for the univariate and multivariate analyses. The odds ratios (ORs) and 95% confidence intervals (95% CIs) were also calculated. All *p* values less than 0.05 according to a two-tailed test were considered significant. Variables that achieved statistical significance (*p* < 0.05) or marginal significance (*p* < 0.10) in the univariate analysis were entered into the multiple logistic regression analysis to identify significant independent factors. All statistical tests were performed with the Statistical Package for Social Sciences software (SPSS Inc., Chicago, IL).

## Results

### Clinical factors associated with TERT C228T-positive liver cancer

Data from all 258 patients who were confirmed to have a diagnosis of liver cancer with imaging studies were analyzed to identify clinical factors associated with TERT C228T-positive liver cancer. The univariate analysis identified 2 parameters that tended to be or were significantly correlated with TERT C228T-positive liver cancer: body mass index (≥ 25.0 kg/m^2^, *p* = 0.047) and etiology (FLD vs. HBV; *p* = 0.009). Biochemical markers reflecting inflammation were not different according to TERT C228T (AST, *p* = 0.194; ALT, *p* = 0.507; Total bilirubin, *p* = 0.551; and AFP, *p* = 0.150; Mann–Whitney *U* test). The multivariate analysis included these factors and identified etiology (FLD vs. HBV; OR 2.346, *p* = 0.010) as a significant and independent determinant of TERT C228T-positive liver cancer (Table 2).

### Histopathological factors associated with TERT C228T-positive liver cancer

Data from 117 patients who underwent surgical resection and were confirmed to have a diagnosis of liver cancer with histopathological examination were analyzed to identify histopathological factors associated with TERT C228T-positive liver cancer. The univariate analysis identified 2 parameters that tended to be or were significantly correlated with TERT C228T-positive liver cancer: vp (presence, *p* = 0.097) and fibrosis stage (0, 1, 2; *p* = 0.047). The multivariate analysis that included these factors identified vp (presence; OR 2.472, *p* = 0.037) and fibrosis stage (0, 1, 2; OR 3.774, *p* = 0.003) as significant and independent determinants of TERT C228T-positive liver cancer (Table 3).

### Relationships between etiology of liver cancer and TERT C228T/AFP/PIVKAII

**Fig. 1:**
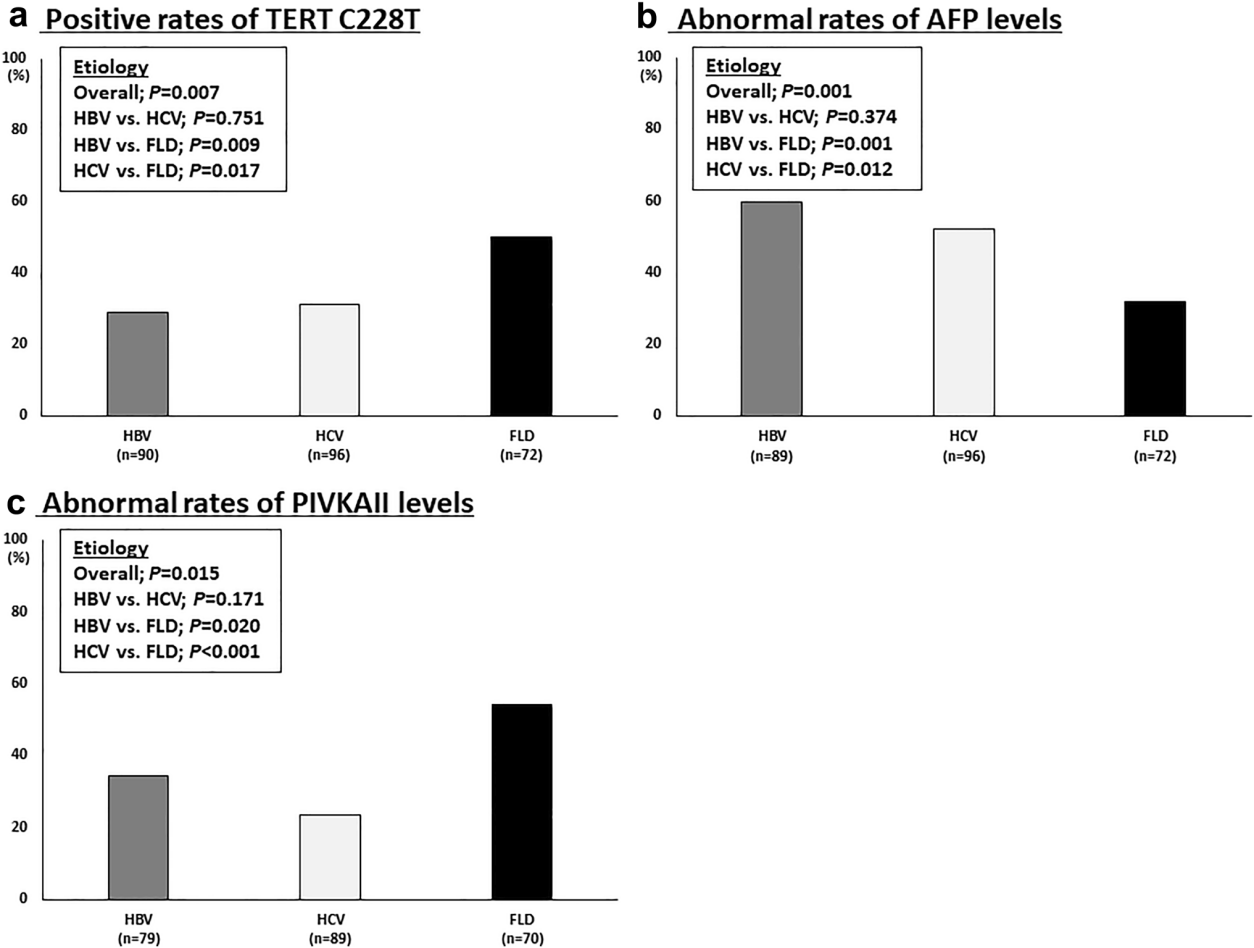
Relationships between etiology of liver cancer and TERT C228T/AFP/PIVKAII. **A** Rates of positive TERT C228T, **B** rates of abnormal AFP levels, and **C** rates of abnormal PIVKAII levels. Normal level of AFP is defined as 10 μg/L or less, and that of PIVKAII is 40 AU/L or less

The rates of positive TERT C228T in FLD patients were significantly higher than those of HBV patients (*p* = 0.009; chi-squared test) and HCV patients (*p* = 0.017; chi-squared test) (Fig. 1A). The rates of abnormal AFP levels in FLD patients were significantly lower than those of HBV patients (*p* = 0.001; chi-squared test) and HCV patients (*p* = 0.012; chi-squared test) (Fig. 1B). The rates of abnormal PIVKAII levels in FLD patients were significantly higher than those of HBV patients (*p* = 0.020; chi-squared test) and HCV patients (*p* < 0.001; chi-squared test) (Fig. 1C). Relationships among etiology, TERT C228T/AFP/PIVKAII, and histopathological findings of 117 patients who underwent liver cancer surgical resection, were shown in Table 4.

**Table 4:** Relationships among etiology, serological markers, and histopathological findings of 117 patients who underwent liver cancer surgical resection

	HBV (*n* = 32)	HCV (*n* = 37)	FLD (*n* = 48)	*p*
Serological markers				
TERT C228T, negative/positive, *n*	23/9	27/10	26/22	0.080
AFP, μg/L	8.5 (1–3706)	10.0 (1–8851)	6.0 (2–16,659)	0.388
PIVKAII, AU/L	40 (1–894)	28 (1–8950)	47 (10–157,050)	0.101
Tumor tissue				
Number of tumors, 1/2/3, *n*	30/2/0	33/3/1	43/5/0	0.642
Maximum tumor diameter, mm	20 (8–47)	20 (4–49)	28 (8–140)	0.001
Organization type				
Well-differentiated HCC, not contain/contain/unknown, *n*	23/7/2	21/16/0	28/20/0	0.137
Moderately-differentiated HCC, not contain/contain/unknown, *n*	3/27/2	5/32/0	4/44/0	0.741
Poorly-differentiated HCC, not contain/contain/unknown, *n*	22/8/2	29/8/0	44/4/0	0.031
Chlangiocellular carcinoma, not contain/contain/unknown, *n*	29/1/2	37/0/0	44/4/0	0.212
Formation of capsule, absence/presence/unknown, *n*	12/14/6	5/31/1	10/38/0	0.040
Infiltration to capsule, absence/presence/unknown, *n*	13/9/10	5/25/7	15/29/4	0.136
Septal formation, absence/presence/unknown, *n*	10/16/6	12/21/4	10/36/2	0.111
Serosal infiltration, absence/presence/unknown, *n*	26/0/6	30/1/6	47/1/0	0.612
Vascular invasion				
vp, absence/presence/unknown, *n*	20/10/2	28/9/0	29/19/0	0.456
vv, absence/presence/unknown, *n*	25/2/5	31/3/3	46/2/0	0.516
va, absence/presence/unknown, *n*	26/0/6	31/0/6	47/0/1	1.000
Bile duct invasion, absence/presence/unknown, *n*	25/0/7	27/0/10	46/2/0	0.183
Intrahepatic metastasis, absence/presence/unknown, *n*	22/1/9	26/0/11	43/2/3	0.832
Peritoneal dissemination, absence/presence/unknown, *n*	16/0/16	24/0/13	41/0/7	1.000
Non tumor tissue				
Steatosis, 5–33% / > 33–66% / > 66%/unknown, *n*^a^	–	–	33/12/2/1	
Lobular inflammation, No foci / < 2 foci/2–4 foci / > 4 foci per 200 × field/unknown, *n*^a^	–	–	2/25/17/2/2	
Ballooning, None/Few cells/Many cells/unknown, *n*^a^	–	–	6/36/4/2	
Fibrosis stage, 0/1/2/3/4/unknown, *n*	1/6/5/4/15/1	0/4/7/5/21/0	0/7/2/22/17/0	0.560

Data are number of patients or median (range) values

*AFP* alpha-fetoprotein, *PIVKAII* des-γ-carboxyprothrombin, *HCC* hepatocellular carcinoma, *HBV* hepatitis B virus, *HCV* hepatitis C virus, *FLD* fatty liver disease

^a^Steatosis, lobular inflammation and ballooning in non tumor tissue were evaluated in patients with FLD

### Relationships between TERT C228T and viral suppression/alcohol intake

**Fig. 2:**
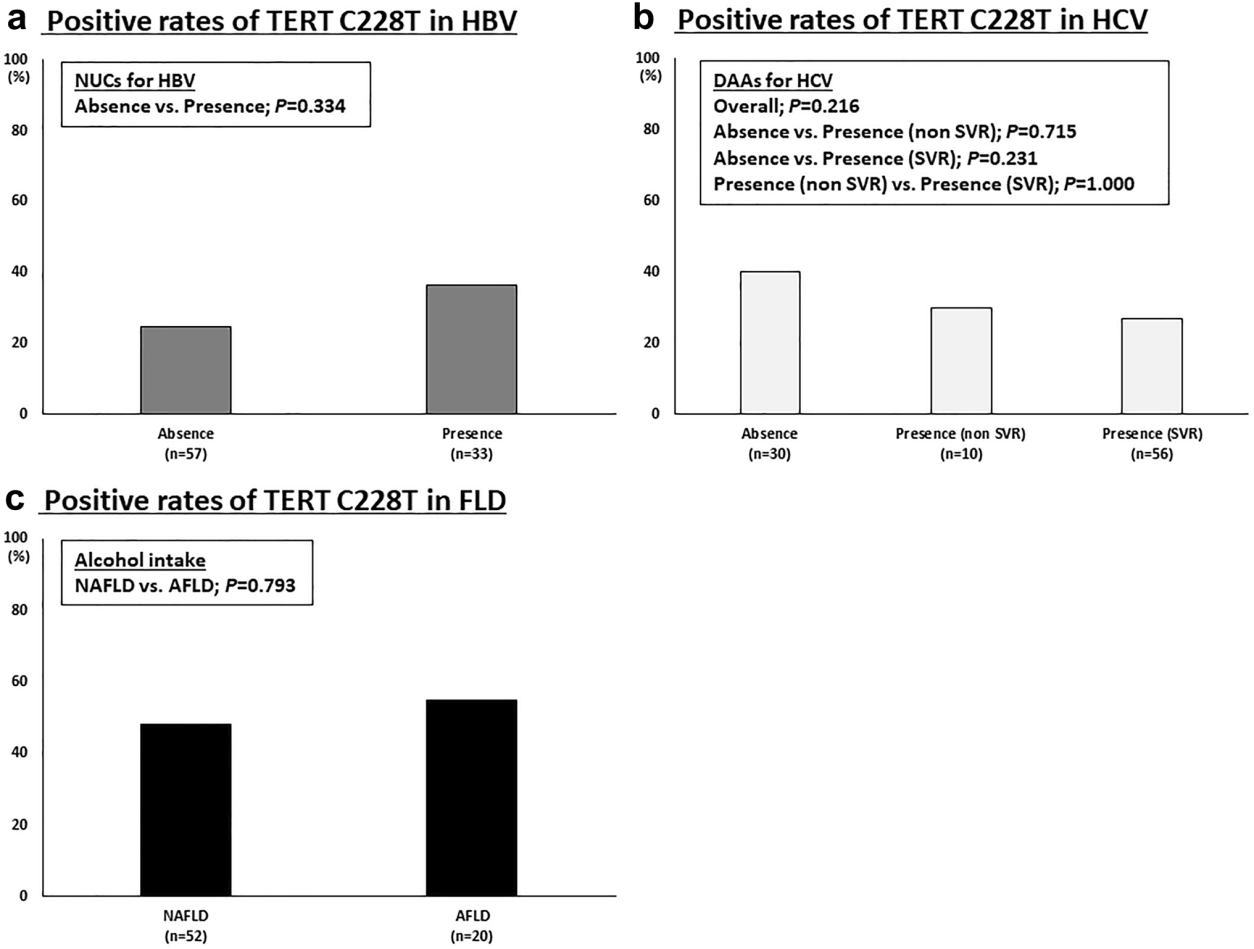
Relationships between TERT C228T and viral suppression/alcohol intake. **A** Rates of positive TERT C228T in HBV, **B** those in HCV, and **C** those in FLD according to viral suppression or alcohol intake. *AFLD* alcoholic fatty liver disease, *DAAs* direct-acting antivirals, *NAFLD* non-alcoholic fatty liver disease, *NUCs* nucleos(t)ide analogues

The rates of positive TERT C228T were not different between the two groups of HBV (57 patients who did not receive NUCs and 33 patients who achieved viral suppression under NUCs) (*p* = 0.334; chi-squared test) (Fig. 2A). There were no differences between the three groups of HCV (30 patients who did not receive DAAs, 10 patients who did not achieve SVR by DAAs, and 56 patients who achieved SVR by DAAs) (*p* = 0.216; chi-squared test) (Fig. 2B). There were also no differences between the two groups of FLD (52 patients who were diagnosed as NAFLD and 20 patients who were diagnosed as AFLD) (*p* = 0.793; chi-squared test) (Fig. 2C). Interestingly, the rates of positive TERT C228T in HCV patients who achieved SVR were significantly lower than those of FLD patients (*p* = 0.011; chi-squared test).

### Relationships between AFP/PIVKAII and viral suppression/alcohol intake

The rates of abnormal AFP levels in HBV patients who did not receive NUCs were significantly higher than those of HBV patients who achieved viral suppression under NUCs (*p* = 0.046; chi-squared test) (Supplement Fig. 1A). There were significant differences between the three groups of HCV (*p* < 0.001; chi-squared test). Particularly, AFP levels of HCV patients who did not receive DAAs were significantly higher than those of HCV patients who achieved SVR by DAAs (*p* = 0.001; chi-squared test) (Supplement Fig. 1B). There were no differences between the two groups of FLD (NAFLD and AFLD) (*p* = 0.575; chi-squared test) (Supplement Fig. 1C).

The rates of abnormal PIVKAII levels in HBV patients showed no differences between patients who did not receive NUCs and patients who achieved viral suppression under NUCs (*p* = 0.636; chi-squared test) (Supplement Fig. 2A). There were no differences between the three groups of HCV (*p* = 0.117; chi-squared test) (Supplement Fig. 2B). There were no differences between the two groups of FLD (NAFLD and AFLD) (*p* = 0.603; chi-squared test) (Supplement Fig. 2C).

## Discussion

Molecular therapies and precision medicine for liver cancer are anticipated. Llovet et al. presented an integrative molecular and immunological classification of liver cancer [13, 14]. From the perspective of etiology, proliferation-class tumors are associated with HBV-related liver cancer, and non-proliferation-class tumors are associated with alcohol and HCV-related liver cancer. HBV-related liver cancer tends to contain the histological features of poorly differentiated HCC and higher frequencies of vascular invasion. Furthermore, HBV-related liver cancer indicates higher AFP levels [13]. Unfortunately, our results could highlight the importance of serum TERT C228T for the detection of non-viral FLD-related liver cancer, but the superiority of TERT C228T could not be compared with AFP or with PIVKAII. Hence, we should require attention to interpretation of the present findings. Further study should be performed to compare the usefulness of three serological markers for the detection of FLD-related liver cancer.

From the perspective of DNA somatic alterations, it is unclear whether there might be a difference among HCV, NAFLD, and alcoholic-related liver cancer. Pinyol et al. reported that rates of positive TERT C228T in NAFLD-related liver cancer were not significantly different from those of HBV/HCV/alcoholic-related liver cancer [15]. However, it is unknown whether the rates of positive TERT C228T might be different among the three etiologies of HBV, HCV, and FLD-related liver cancer. The present results indicated that those of FLD were significantly higher than those of HBV and HCV, while those of NAFLD were not different from those of AFLD. To our knowledge, the present study is the first to highlight the importance of TERT C228T for the detection of non-viral FLD-related liver cancer. The present findings based on the difference of etiology might be useful for the development of molecular therapies and precision medicine for liver cancer. As one limitation of the present study, we could not examine TERT C228T in the precancerous serum samples without liver cancer. Our previous report showed the rates of positive TERT C228T were 4.8% in serum samples of NAFLD without liver cancer (Supplement Table) [8]. Other previous report indicated positive rates of 8.6% in plasma samples of cirrhosis without liver cancer, including the etiologies of HBV, HCV, and FLD [16]. Further study according to the etiology should be performed to investigate the difference in the rates of positive TERT C228T in precancerous stage without liver cancer.

Pfister et al. recently reported that NAFLD-related liver cancer might be less responsive to immune checkpoint inhibitors, which is probably due to NAFLD-related aberrant T cell activation causing tissue damage that leads to impaired immune surveillance [6]. Compared to other etiologies, NAFLD-related liver cancer shows a significantly higher prevalence of an immunosuppressive cancer field [15]. This evidence provides a rationale for stratification of patients with liver cancer according to the underlying etiology in studies of immunotherapy as a primary or adjuvant treatment. One limitation of the present study is the lack of analysis of comparison between tissue of liver cancer and the corresponding cfDNA. Previous report indicated that *TERT* promoter mutations in tissue of cirrhosis correlate with the rate of hepatocarcinogenesis, with mutations identified in 6% of low grade dysplastic nodules, 19% of high grade dysplastic nodules, and 61% of early liver cancer [17]. However, it is still unclear whether mutations of cfDNA might reflect those of tissue. Another limitation of the present study is that the difference in *Wnt*/*CTNNB1* mutations, apart from *TERT* promoter mutations, could not be investigated according to the etiology of liver cancer. Further studies should be performed to develop molecular therapies and precision medicine for liver cancer based on DNA somatic alterations.

To our knowledge, the present study is the first to investigate the relationships between TERT C228T and viral suppression/alcohol intake. Basically, neither viral suppression nor alcohol intake significantly affected the rates of positive TERT C228T. On the other hand, viral suppression of HBV and HCV significantly reduced AFP levels. One reason for these discrepant results might be that AFP levels reflect not only the potential of carcinogenesis, but also higher levels of inflammation [18–20]. Hence, the present results also showed that TERT C228T was a tumor marker that might not be influenced by inflammation. Interestingly, the rates of positive TERT C228T in HCV patients who achieved viral clearance were significantly lower than those of FLD patients. This finding indicates that the two groups of FLD and HCV with SVR should be classified in precision medicine for liver cancer based on DNA somatic alterations.

In conclusion, our results highlight the importance of serum TERT C228T for the detection of non-viral FLD-related liver cancer. TERT C228T is a tumor marker that might not be influenced by inflammation. Early diagnosis and treatment based on DNA somatic alterations might improve the outcome of non-viral FLD patients who develop liver cancer.

## Supplementary Information

Below is the link to the electronic supplementary material.

Supplement Figure 1. Relationships between AFP levels and viral suppression/alcohol intake. (A) Rates of abnormal AFP levels in HBV, (B) those in HCV, and (C) those in FLD according to viral suppression or alcohol intake. Normal level of AFP is defined as 10 μg/L or less. AFLD: alcoholic fatty liver disease, DAAs: direct-acting antivirals, NAFLD: non-alcoholic fatty liver disease: NUCs: nucleos(t)ide analogues

Supplement Figure 2. Relationships between PIVKAII levels and viral suppression/alcohol intake. (A) Rates of abnormal PIVKAII levels in HBV, (B) those in HCV, and (C) those in FLD according to viral suppression or alcohol intake. Normal level of PIVKAII is 40 AU/L or less. AFLD: alcoholic fatty liver disease, DAAs: direct-acting antivirals, NAFLD: non-alcoholic fatty liver disease: NUCs: nucleos(t)ide analogues

Supplementary file3 (DOCX 17 KB)

## Data Availability

The datasets generated or analyzed in the present study are available from the corresponding author on reasonable request.

## Abbreviations

AFLD: Alcoholic fatty liver disease
AFP: Alpha-fetoprotein
ALT: Alanine aminotransferase
AST: Aspartate aminotransferase
BCLC: Barcelona Clinic Liver Cancer
CCC: Cholangiocellular carcinoma
cfDNA: Cell-free DNA
DAAs: Direct acting antivirals
FLD: Fatty liver disease
NAFLD: Non-alcoholic fatty liver disease
NASH: Non-alcoholic steatohepatitis
HBV: Hepatitis B virus
HCC: Hepatocellular carcinoma
HCV: Hepatitis C virus
LNA: Locked nucleic acid
MT: Mutant-type
NUCs: Nucleos(t)ide analogues
PCR: Polymerase chain reaction
PIVKAII: Des-γ-carboxyprothrombin
SVR: Sustained virological response
*TERT*: Telomerase reverse transcriptase
WTB: Wild-type blocking
