# Supplementary figures and images for: Serum TERT C228T is an important predictor of non-viral liver cancer with fatty liver disease
Source: Hepatol Int. 2022 Mar 20;16(2):412–22. doi: 10.1007/s12072-022-10313-y (PMC9013341; doi:10.1007/s12072-022-10313-y)

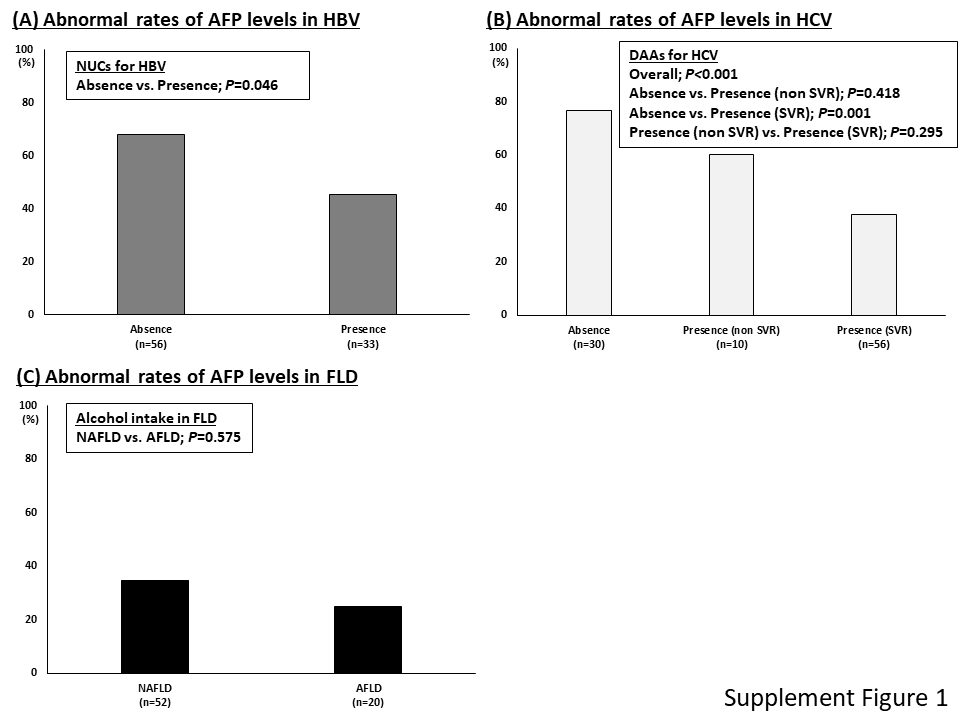

Supplement: Supplementary file 1 — Supplement Figure 1. Relationships between AFP levels and viral suppression/alcohol intake. (A) Rates of abnormal AFP levels in HBV, (B) those in HCV, and (C) those in FLD according to viral suppression or alcohol intake. Normal level of AFP is defined as 10 μg/L or less. AFLD: alcoholic fatty liver disease, DAAs: direct-acting antivirals, NAFLD: non-alcoholic fatty liver disease: NUCs: nucleos(t)ide analogues [file 12072_2022_10313_MOESM1_ESM.tif]

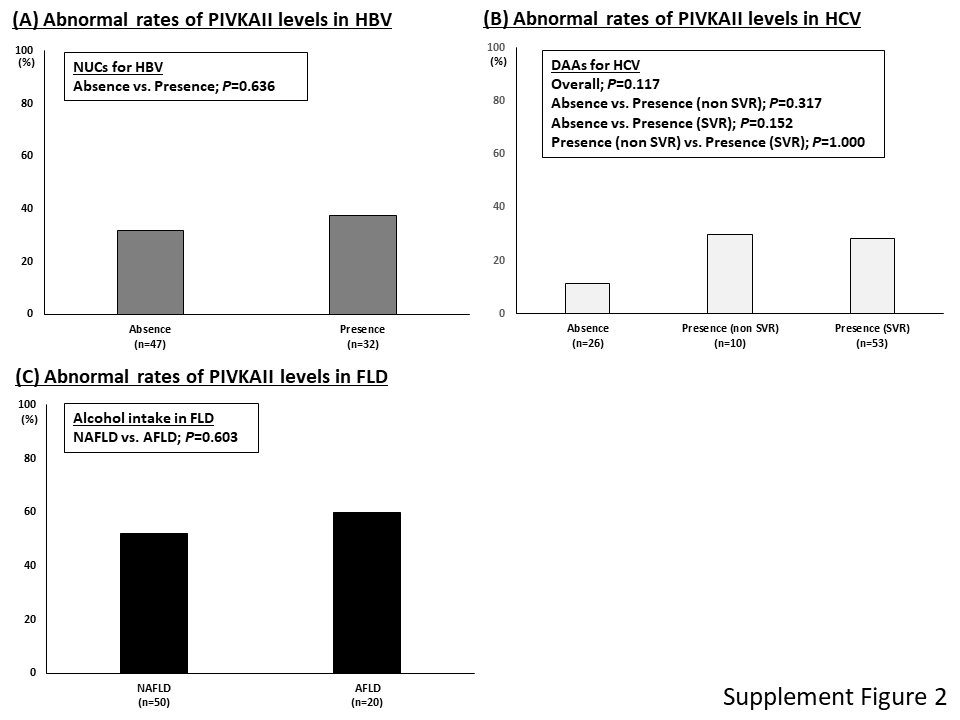

Supplement: Supplementary file 2 — Supplement Figure 2. Relationships between PIVKAII levels and viral suppression/alcohol intake. (A) Rates of abnormal PIVKAII levels in HBV, (B) those in HCV, and (C) those in FLD according to viral suppression or alcohol intake. Normal level of PIVKAII is 40 AU/L or less. AFLD: alcoholic fatty liver disease, DAAs: direct-acting antivirals, NAFLD: non-alcoholic fatty liver disease: NUCs: nucleos(t)ide analogues [file 12072_2022_10313_MOESM2_ESM.tif]
