# Supplementary material for: Serum TERT C228T is an important predictor of non-viral liver cancer with fatty liver disease
Source: Hepatol Int. 2022 Mar 20;16(2):412–22. doi: 10.1007/s12072-022-10313-y (PMC9013341; doi:10.1007/s12072-022-10313-y)
Supplement: Supplementary file 3 — Supplementary file3 (DOCX 17 KB) [file 12072_2022_10313_MOESM3_ESM.docx]

| **Supplement Table**  **The present results, based on all patients with liver cancer.** | | |
| --- | --- | --- |
|  | FLD positive (n=72) | FLD negative (n=186) |
| TERT positive (n=92) | 36 | 56 |
| TERT negative (n=166) | 36 | 130 |
|  |  |  |
|  | FLD positive (n=72) | FLD negative (n=185) |
| AFP positive (n=126) | 23 | 103 |
| AFP negative (n=131) | 49 | 82 |
|  |  |  |
|  | FLD positive (n=70) | FLD negative (n=168) |
| PIVKAII positive (n=86) | 38 | 48 |
| PIVKAII negative (n=152) | 32 | 120 |
|  |  |  |
| **Our previous report, based on NAFLD patients with or without liver cancer.(Oncology 2021;99:114-123)** | | |
|  | Presence of Liver cancer (n=36) | Absence of Liver cancer (n=21) |
| TERT positive (n=24) | 23 | 1 |
| TERT negative (n=33) | 13 | 20 |
|  |  |  |
|  | Presence of Liver cancer (n=36) | Absence of Liver cancer (n=21) |
| AFP positive (n=20) | 13 | 7 |
| AFP negative (n=37) | 23 | 14 |
|  |  |  |
|  | Presence of Liver cancer (n=34) | Absence of Liver cancer (n=19) |
| PIVKAII positive (n=16) | 16 | 0 |
| PIVKAII negative (n=37) | 18 | 19 |
|  |  |  |
